# Long-term effects of different hypoglycemic drugs on carotid intima-media thickness progression: a systematic review and network meta-analysis

**DOI:** 10.3389/fendo.2024.1403606

**Published:** 2024-05-31

**Authors:** Qianyu Lv, Yingtian Yang, Yanfei Lv, Qian Wu, Xinzheng Hou, Lanlan Li, Xuejiao Ye, Chenyan Yang, Shihan Wang

**Affiliations:** ^1^ Guang’anmen Hospital, China Academy of Chinese Medical Sciences, Beijing, China; ^2^ School of Management, Fudan University, Shanghai, China

**Keywords:** atherosclerosis, intima-media thickness, antidiabetic drug, cardiovascular, diabetes

## Abstract

**Objective:**

The progression of carotid intima-media thickness (cIMT) can partially predict the occurrence of future cardiovascular events. This network meta-analysis compared the effects of 14 antidiabetic drugs (acarbose, alogliptin, exenatide, glibenclamide, glimepiride, ipragliflozin, metformin, nateglinide, pioglitazone, rosiglitazone, sitagliptin, tofoglifozin, troglitazone, voglibose) on the progression of cIMT.

**Method:**

PubMed, EMBASE, Cochrane Library, and Web of Science were searched to screen all clinical trials of treatment of cIMT with hypoglycemic agents before March 1, 2024. The differences in the changes in cIMT between the treatment group and control group were evaluated.

**Result:**

After screening 8395 citations, 25 studies (6675 patients) were included. The results indicated that exenatide had the best efficacy in slowing down cIMT progress, and exenatide [MD=-0.13,95%CI (-0.25, -0.01)], alogliptin [MD=-0.08,95%CI (-0.13, -0.02)] and metformin [MD=-0.05, 95%CI (-0.09, -0.02)] are more effective than placebo.

**Conclusion:**

Long-term treatment of exenatide, alogliptin, and metformin may be more effective than other hypoglycemic drugs in slowing the progression of cIMT.

**Systematic Review Registration:**

https://www.crd.york.ac.uk/PROSPERO/, identifier CRD42024519474.

## Introduction

Atherosclerosis (AS) plays a crucial role in the pathogenesis of cardiovascular diseases, which may ultimately lead to atherosclerotic cardiovascular disease (ASCVD), posing significant threats to human health. In this pathological process of atherosclerosis formation, excessive lipid deposition and macrophage transformation into foam cells lead to the increase in intima-media thickness (IMT) (1). In fact, IMT can assess the extent of atherosclerosis in the whole human body (2), especially the carotid intima-media thickness (cIMT), serves as a crucial indicator for cardiovascular disease, whose progression can predict future cardiovascular events to a certain extent (3–5). Dysglycemia is generally considered to be an important risk factor for AS (6). Some traditional hypoglycemic drugs(HD) have shown good cardiovascular safety (7–9), especially the clinical application of novel types of hypoglycemic drugs, including dipeptidyl peptidase-4 inhibitor (DPP-4i), glucagon like peptide-1 receptor agonists (GLP-1 RA), sodium-glucose cotransporter-2 inhibitor (SGLT-2i), peroxisome proliferator-activated receptor gamma (PPARγ) agonist, has ushered in a new era of diabetes treatment. These drugs are increasingly recognized to have multiple effects beyond lowering blood glucose, some of which may contribute to clinical practice in the treatment of cardiovascular diseases (10–13), SGLT-2i and GLP-1 RA are also included in the guidelines as a recommended drug for patients with diabetes and at high risk for cardiovascular events (14, 15). Some clinical trials have conducted to explore the efficacy of one or more HDs and reported the changes of cIMT, however, the impact of different HDs on cIMT has not yet been identified. A network meta-analysis (NMA) can simultaneously compare the effects of multiple interventions because of the lack of direct comparisons between these drugs (16). Therefore, we extracted data from relevant studies to conduct a NMA to assess the relative effectiveness of different HDs in cIMT changes, and to provide evidence for clinical application.

## Methods

### Study design and registration

We developed a detailed search strategy following Preferred Reporting Items for Systematic Reviews and Meta-Analyses (PRISMA) guidelines (17), the research protocol was registered with PROSPERO(CRD42024519474).

### Study selection

A comprehensive search was conducted using four databases (PubMed, Embase, Cochrane Library, and Web of Science), for all studies on the relationship between HDs and cIMT. The search strategy was implemented by multiplying the search formulas for HDs and carotid intima-media thickness/atherosclerosis. The search period was from the establishment of the database to March 1, 2024. The complete search strategy is given in additional file: **Appendix S1**.

### Inclusion and exclusion criteria

Identified studies were enrolled based on the following inclusion criteria: (a) to evaluate the therapeutic effect of various HDs in retarding cIMT; (b) recruited patients with Type 1 diabetes mellitus(T1DM), type 2 diabetes mellitus(T2DM) or impaired glucose tolerance (IGT) or impaired fasting glucose(IFG) or coronary heart disease (CVD) or metabolic syndrome (MS) or abnormal glucose metabolism(AGM);(c) recruited patients were at least 18 years of age; (d) the duration of treatment is at least one year; (e) had a placebo (PLC) or comparative drug control group to compare the outcomes.

Studies containing the following items were excluded: (a) was not a randomized controlled trial (RCT) trial; (b) a combination of drugs was included in the treatment or control group; (c) treatment duration is less than one year; (d) the study did not provide sufficient information about the dataset; (e) animal experiments, review, protocol, case report, *post hoc* analysis; (f) non-English language literature.

### Data extraction and quality assessment

Firstly, the possibly relevant research was imported into EndNote X9, after removing duplicates, independent literature data extraction is conducted by two reviewers, the title and abstract are reviewed to exclude unqualified literature, and then the full text evaluation is conducted. This NMA mainly extracted the basic information of the included literature (name and country of the main author, publication year), basic characteristics of the study population (number of cases, age, sex, disease situation, basic treatment), and outcome indicators (changes of the cIMT).

Literature quality was assessed using the RoB (18) in the following five bias domains: randomization process, allocation concealment, intervention blinding of participants and investigators, missing outcome data, outcome measurement and selection of reported results. An algorithm was employed to estimate the overall risk of bias, i.e., low risk, some concerns, or high risk. The above bias risk assessment was independently conducted and cross-checked by two reviewers. In case of disagreement, a third reviewer was involved to discuss and determine the evaluation result.

### Data analysis

The statistical model of Bayesian framework was constructed using JAGS software 4.3.1 (gemtc package 0.8–2 and rjags package 4–10) (Rstudio, Boston, MA, USA), cIMT(mm) was used as continuous variable, while mean difference (MD) and 95% credible interval (CI) were used as effect indexes. A random effects model with four Markov chains, each chain generating 50,000 iterations (burn-in period of 20,000 iterations). was used for the outcome indicator. The convergence of iterations was monitored using plots and the Gelman–Rubin–Brooks statistic (19). Model consistency was assessed using the deviation information criterion (DIC), with differences in DIC < 5 points indicating good consistency and consistency modeling was used (20). Heterogeneity was estimated using the I^2^ statistic with I^2^ values < 25% indicating low heterogeneity, 25 to 75%, moderate heterogeneity, and > 75%, high heterogeneity (21). Publication bias was assessed by funnel plots. Network plots and funnel plots were drawn by Stata SE 15.0 (Stata Corp, College Station, Texas, USA). In addition, we also comparing the therapeutic effect of HDs using a Surface Under the Cumulative Ranking curve (SUCRA) (22), the closer SUCRA was to 100, the better the therapeutic effect. What is more, we performed sensitivity analyses to examine the influence of individual studies on the total merged effects to assess the stability of the conclusion, and publication bias was analyzed by funnel plot using Stata16.0 software.

## Results

### Data characteristics

**Figure 1:**
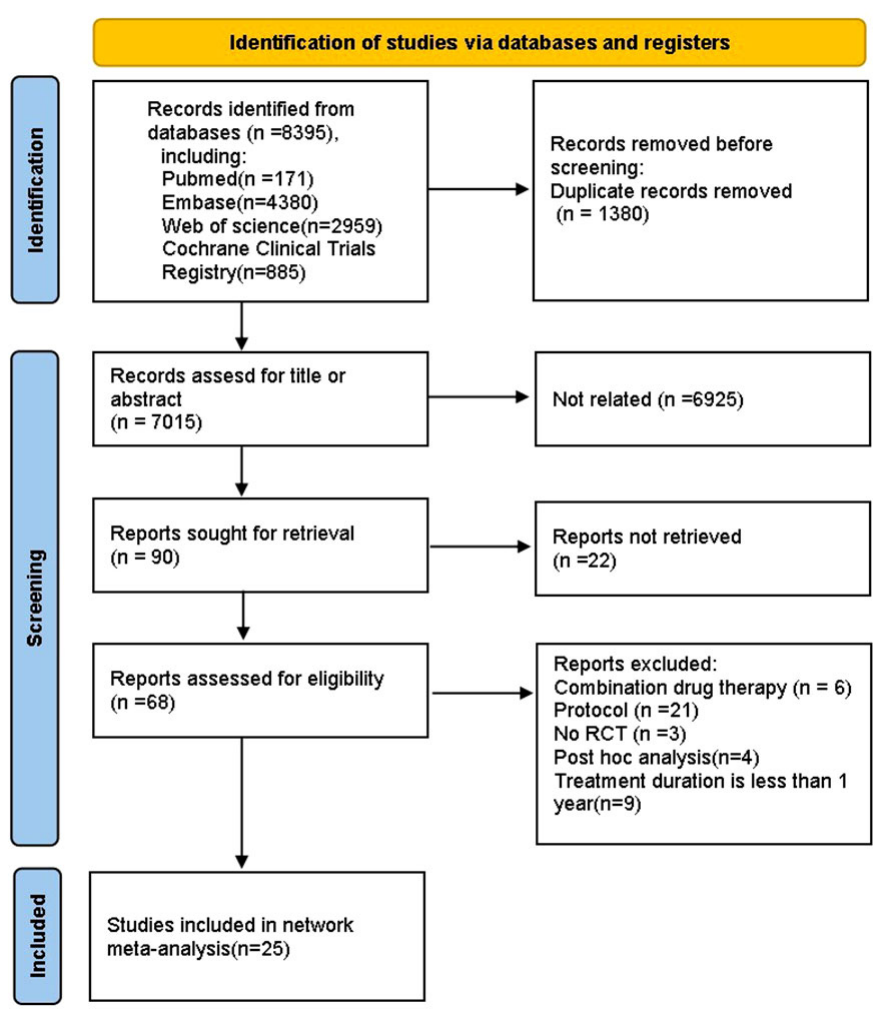
Flow chart of literature screening in the NMA.

We finally identified 8395 studies through an extensive search, after removing 1380 duplicates, we reviewed the titles and abstracts of the remaining articles based on eligibility criteria. 6959 studies were excluded because they were not relevant to our NMA, original articles were not found for 22 articles, 6 papers reported the combined use of drugs, 21 protocols, 3 studies were not RCT, 4 were *post hoc* analyses, 9 studies had a treatment duration less than one year. Ultimately, 25 studies (12, 23–46) were included in the NMA, and the literature screening process is shown in the (**Figure 1**). Our NMA included 14 HDs (acarbose, alogliptin, exenatide, glibenclamide, glimepiride, ipragliflozin, metformin, nateglinide, pioglitazone, rosiglitazone, sitagliptin, tofoglifozin, troglitazone, voglibose and PLC). A total of 6720 patients with complete outcome data were included in the NMA. Among them, there were 2491 patients with pre-diabetes, while the number of patients with diabetes was 3980. The main basic characteristics of the included patients are shown in **Table 1**.

**Table 1:** Main basic characteristics of the included patient.

Author(year)	Country	Size	Male(%)	Age	Drug vs. Comparator	Duration(year)	Study population
Hanefeld et al. (23)	Germany	115	70(60.8)	55.3 ± 7	Acarbose vs.PLC	3.9	IGT
Nakamura et al. (24)	Japan	45	25(55.6)	64.6 ± 9.5	Pioglitazone vs. Glibenclamide vs.Voglibose	1	T2DM
Sidhu et al. (25)	England	92	41(85.9)	62.5 ± 8	Rosiglitazone vs. PLC	1	CVD
Xiang et al. (26)	America	192	0(0)	34.6 ± 6.8	Troglitazone vs. PLC	2	GDM history
Hodis et al. (27)	America	276	230(67.4)	52.5 ± 9	Troglitazonevs. PLC	2	T2DM
Mazzone et al. (28)	America	361	111 (63.7)	66.3 ± 8	Pioglitazone vs. Glimepiride	1.5	T2DM
Hedblad et al. (29)	England	555	255(45.9)	66.5 ± 7	Rosiglitazone vs. PLC	1.1	T2DM
Mita et al. (30)	Japan	70	33(47.1)	61.5 ± 7	Nateglinide vs. PLC	1	T2DM
Lonn et al. (32)	Canada	1425	638(44.78)	53.9 ± 10.8	Rosiglitazone vs. PLC	1	IFG
Koyasu et al. (33)	Japan	81	74 (91.4)	66.3 ± 8	Acarbose vs. PLC	1	CVD
Yamasaki et al. (35)	Japan	186	117(62.9)	56.9	Pioglitazone vs. PLC	4	T2DM
Yasunari et al. (34)	Japan	48	12(25)	56.7 ± 10.4	Pioglitazone vs. PLC	4	T2DM
Saremi et al. (36)	America	382	175(45.8)	53.5 ± 11.5	Pioglitazone vs. PLC	2.3	IGT
Patel et al. (37)	America	219	74(33.8)	53.6 ± 11	Acarbose vs. PLC	5	IFG
Ishikawa et al. (38)	Japan	76	65(85.5)	71.4 ± 8	Sitagliptin vs. PLC	2.2	CVD
Mita et al. (39)	Japan	341	199(58.4)	64.6 ± 9.5	Alogliptin vs. PLC	2.2	T2DM
Mita et al. (39)	Japan	282	165 (60.2)	63.7 ± 9.8	Sitagliptin vs. PLC	2.2	T2DM
Oyama et al. (41)	Japan	442	297 (67.2)	69.3 ± 9	Sitagliptin vs. PLC	2	T2DM
Katakami et al. (42)	Japan	339	198(58.4%)	61.1 ± 9.5	Tofoglifozin vs. PLC	2.2	T2DM
Zhang et al. (43)	China	59	33(55.9)	58.4 ± 12.8	Exenatide vs. PLC	1.1	T2DM
Oyama et al. (31)	Japan	84	30(35.7)	60 ± 5	Acarbose vs. PLC	1	T2DM
Tanaka et al. (12)	Japan	464	161 (69.4)	68 ± 5.9	Ipragliflozin vs. PLC	2	T2DM+CVD
Meaney et al. (44)	Mexico	58	17(29.3)	49 ± 9	Metformin vs. PLC	1	MS
Petrie et al. (45)	England	428	175(40.89%)	55.5 ± 8.6	Metformin vs.Acarbose	3	T1DM
Lin et al. (46)	China	100	58(58%)	68.1 ± 7.8	Metformin vs.Acarbose	1	AGM

AGM, coronary heart disease; CVD, coronary heart disease; GDM, gestational diabetes mellitus; IFG, impaired fasting glucose; IGT, impaired glucose tolerance; MS, metabolic syndrome; T1DM, Type 2 diabetes mellitus; T2DM, Type 2 diabetes mellitus.

### Quality assessment

**Figure 2:**
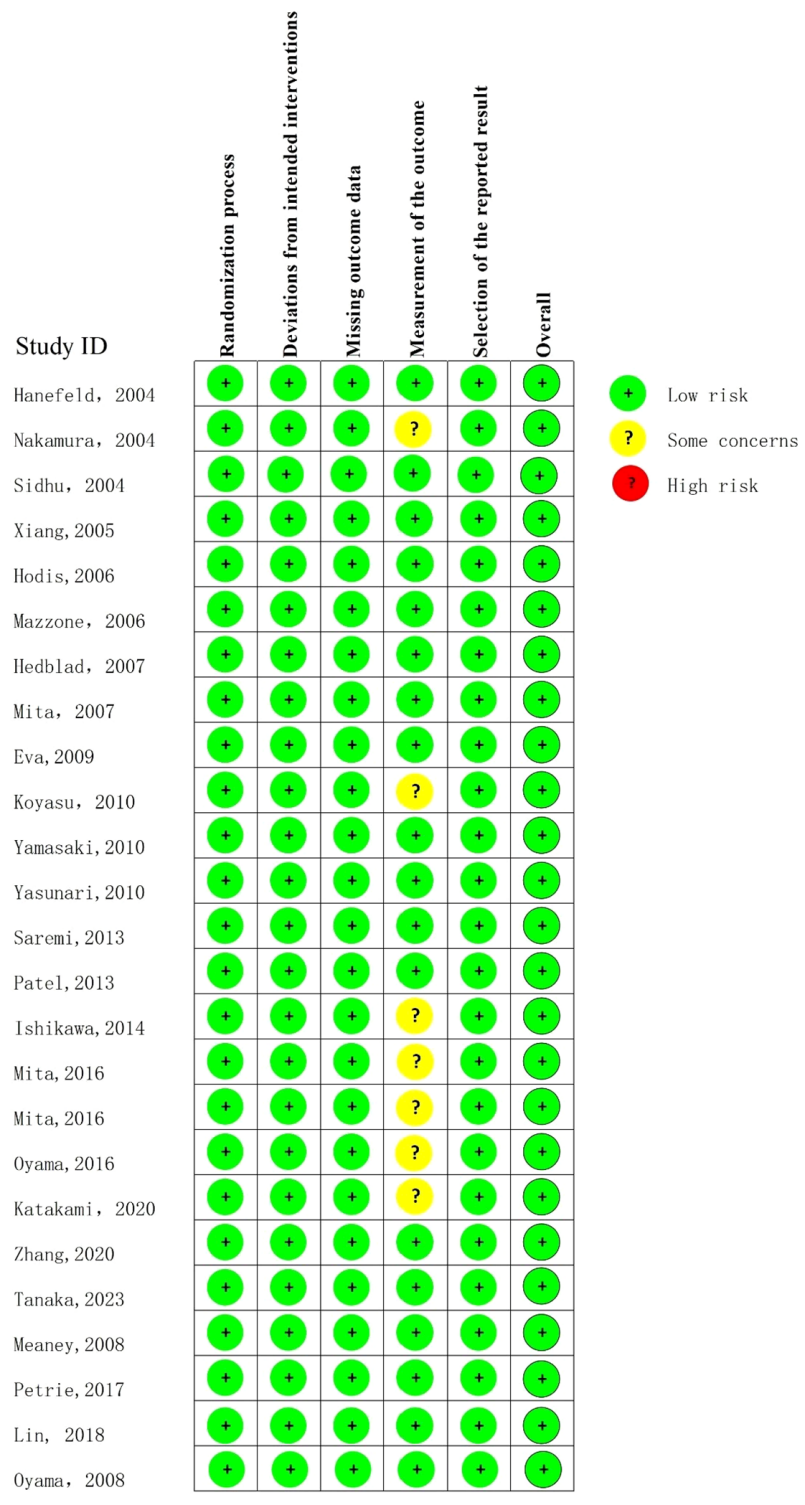
Risk of bias traffic light plot of ROB 2 assessments.

The quality assessment results showed that the overall quality of the included literatures was high (**Figure 2**).

### NMA outcomes

**Figure 3:**
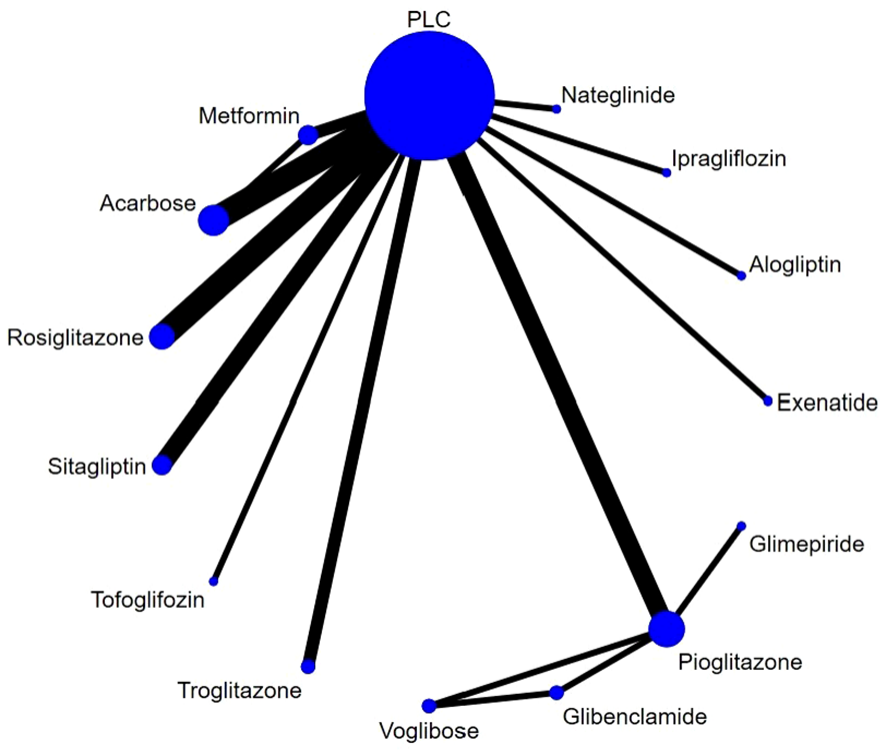
Network plot of clinical trials on HDs or PLC patients. Nodes stand for the comparison between treatments and the size is proportional to the number of subjects. The width of the lines is proportional to the number of trials per pair of interventions.

**Figure 4:**
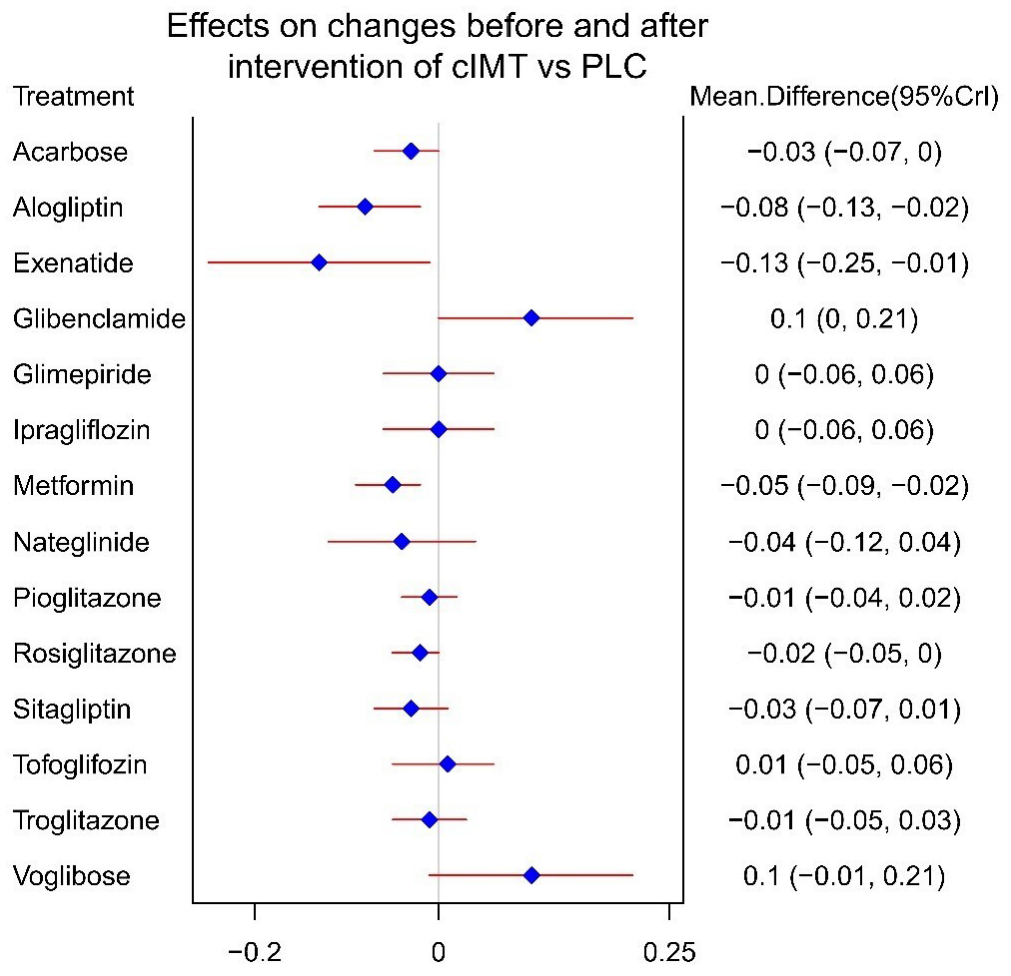
Forest plot showing the outcomes of NMA (relative differences of various HDs in reducing cIMT compared with PLC).

The NMA network diagram involved conducting direct and indirect comparisons among a total of 14 drugs (**Figure 3**), DIC comparison results showed good agreement (DIC, 102.6 VS. 103.0). According to the NMA, exenatide [MD=-0.13,95%CI (-0.25, -0.01)] was found to be the most promising drug for slowing the progression of cIMT comparing to PLC, followed by alogliptin [MD=-0.08,95%CI (-0.13, -0.02)] and metformin [MD=-0.05, 95%CI (-0.09, -0.02)], the forest diagram is shown in **Figure 4**.

What’s more, the results of our pairwise comparison analysis among HDs indicate that exenatide[MD=-0.23, 95%CI (-0.39, -0.08)], alogliptin[MD=-0.18,95%CI (-0.3, -0.06)], metformin[MD=-0.16, 95%CI (-0.27, -0.05)], nateglinide[MD=-0.14,95%CI (-0.27, -0.01), pioglitazone[MD=-0.11, 95%CI (-0.21, -0.01)], rosiglitazone[MD=-0.12, 95%CI (-0.23, -0.02)], sitagliptin[MD=-0.13, 95%CI (-0.24, -0.02)] was more effective than glibenclamide; exenatide[MD=-0.14, 95%CI (-0.27, -0.01)] and alogliptin[MD= -0.08, 95%CI (-0.16, -0.01)] was superior to that of tofoglifozin. However, voglibose demonstrated inferior efficacy compared to exenatide [MD=0.23, 95%CI (0.39, 0.07), alogliptine[MD=0.17, 95%CI (0.3, 0.05)], metformin [MD=0.15, 95%CI (0.27, 0.04)] and sitagliptin[MD=0.13, 95%CI (0.24, 0.01)].

The drug with the highest SUCRA value was exenatide(SUCRA 94.9%), followed by alogliptin(SUCRA 86.9%) and metformin(SUCRA 81.2%), PLC has a SUCRA values with 31.7%, **Table 2** shows the results of the SUCRA analysis.

**Table 2:** The rank of various of HDs and PLC on retarding the progression cIMT.

Treatment	SUCRA	RANK	Treatment	SUCRA	RANK
Exenatide	94.9%	1	Troglitazone	43.7%	9
Alogliptin	86.9%	2	Ipragliflozin	36.6	10
Metformin	81.2%	3	Glimepiride	35.3	11
Nateglinide	67.1%	4	PLC	31.8	12
Acarbose	63.1	5	Tofoglifozin	29.7	13
Sitagliptin	62.8	6	Voglibose	6.5%	14
Rosiglitazone	56.9%	7	Glibenclamide	5.1%	15
Pioglitazone	45.5%	8	–	–	–

### Sensitivity analysis

A sensitivity analyses was conducted by systematically excluding individual studies and performing an additional meta-analysis with each study removed. The impact of each exclusion on the pooled MD was evaluated. Based on the results of the sensitivity analysis, we found that none of the studies significantly influenced the overall effect, indicating the robustness of our NMA. Results of sensitivity analyses are provided in **Appendix S2**.

### Bias of publication

**Figure 5:**
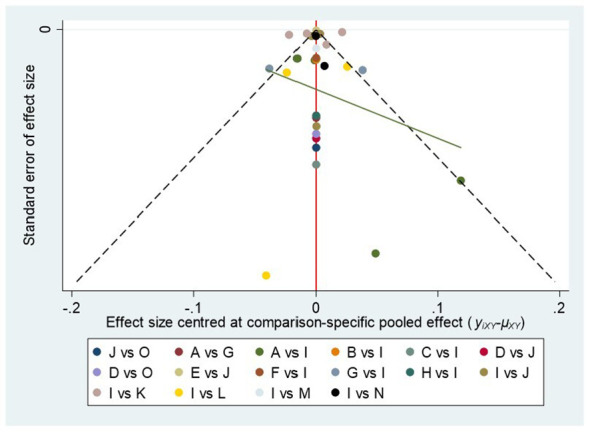
Plot of funnel (A, acarbose; B, alogliptin; C, exenatide; D, glibenclamide; E, glimepiride; F, ipragliflozin; G, metformin; H, nateglinide; I, PLC; J, pioglitazone; K, rosiglitaxone; L, sitagliptin; M, tofoglifozin; N, roglitaxone; O, voglibose).

There was no significant publication bias in the funnel plot of bias analysis drawn for cIMT (**Figure 5**).

## Discussion

The presence of abnormal blood glucose levels significantly exacerbates the morbidity and mortality associated with atherosclerosis (47, 48). It is of great interest to reduce the occurrence of cardiovascular events through controlling the risk factors. We compared the treatment effects of long-term usage of 14 types of HD on cIMT in individuals with and at high risk for diabetes in our NMA. The results of our NMA indicate that exenatide may be the optimal treatment option, followed by alogliptin and metformin.

Exenatide is a short-acting analogue of the Glucagon-like peptide-1(GLP-1). This type of medications worked by binding to the GLP-1 receptor, improving insulin sensitivity and insulin secretion function, and help to lower blood glucose levels (49–51). Recently, it has been discovered that the utilization of GLP-1 RA can not only effectively regulates blood glucose levels but also exerts significant effects on the modulation of blood lipids, blood pressure, and adipose content (52–54), all of which are recognized risk factors for AS (55–57). Thus, he protective effect of GLP-1 RA on the cardiovascular system may, in part, be attributed to its capacity for mitigating these risk factors. A large meta-analysis, involving 60,080 patients, reported that GLP-1 RA can reduce major adverse cardiovascular events (MACE) in type 2 diabetes patients with ASCVD by 14%, and lowers the risk of hospitalization due to heart failure (58). Our NMA suggests that exenatide, among the 14 drugs included, may be the best treatment for retarding the progression of cIMT. Dyslipidemia has been demonstrated to be a significant risk factor for the development of AS. Various studies have shown that exenatide significantly reduces serum total cholesterol(TC), triglyceride (TG), and low-density lipoprotein cholesterol(LDL-C) levels while increasing high density lipoprotein cholesterol (HDL-C) levels in patients with T2DM, and exhibiting favorable long-term effects (59–61). Koska et al. reported that once weekly administration of exenatide resulted in positive effects on blood glucose and body weight, as well as an appropriate increase in heart rate, which is also a risk factor for carotid atherosclerosis (62, 63). Several *in vivo* experiments suggest the potential mechanism by which exenatide improves IMT. Yang et al. (64) found that exenatide could inhibit oxidative stress and inflammation in mice, and improve the accumulation of plaque macrophages and osteopontin expression. Jansen et al. (65) demonstrated that exenatide improved abdominal fat deposition in high-fat fed mice. At the same time, there was study reported no statistically significant difference in the incidence of major adverse cardiovascular events with once-weekly exenatide versus PLC (66).

Only one GLP-A RA, exenatide, was included in our study. However, at the same time, some other types of GLP-A RA have also attracted our attention because of their cardiovascular protective effects (67, 68). Related to our study, a real-world study in patients with T2DM showed that the addition of liraglutide to metformin monotherapy significantly reduced cIMT, as well as serum TC, TG, and LDL-C (69). Another clinical study reported that 4 months of semaglutide injection treatment could also reduce cIMT to some extent (70). Unfortunately, these studies were not included in the NMA because they lacked a control group.

The NMA findings also indicate that alogliptin, a DDP-4i, achieved good performance in intervening cIMT progression. DPP-4i, belong to the group of incretin-based medications that act by stimulating the insulin secretion and inhibiting glucagon secretion in a glucose-dependent manner (71). A prospective clinical study observed that a 10-month treatment of alogliptin intervention can significantly reduce the volume of AS plaque and necrosis, as well as an increase in fibrosis volume compared to PLC (72). In addition, a mechanistic study had shown that alogliptin can inhibit the expression of inflammatory factors interleukin-1(IL-1) and Interleukin-6(IL-6) through a glucose-dependent or independent way (73). However, the extended study based on the SPEAD-A trial followed T2DM patients for 520 weeks and found that early use of alogliptin was not associated with a reduction in the risk of developing cardiovascular disease, which may be related to a relatively small number of events during follow-up (74). A clinical trial conducted by Barbieri et al. (75) showed that both 3 months of sitagliptin and vildagliptin treatment may possible to prevent AS progression in patients with T2DM by reducing inflammation and oxidative stress, moreover, compared with baseline, vildagliptin was more effective in reducing IMT than sitagliptin. It has also reported that vildagliptin is more effective than sitagliptin in controlling the mean 24-hour blood glucose level at a same dose (76), and vildagliptin also shows a superior effectiveness in improving oxidative stress and inflammatory markers, the variations in effectiveness among these drugs are likely attributed to their structural heterogeneity (77).

Our study also included three PPARγ agonists, pioglitazone, rosiglitazone and troglitazone, but no significant improvement was observed for any of them compared with PLC. Contrary to our study, a previous meta-analysis reported a statistically significant reduction in the progression of cIMT with the use of pioglitazone (78). At the same time, clinical studies and meta-analyses (79, 80) have reported that long-term use of rosiglitazone may increase the risk of cardiovascular mortality. Indeed, the administration of pioglitazone and rosiglitazone was associated with the adverse effect of fluid retention, which potentially contributed to or exacerbated congestive heart failure (81).

Despite numerous reports demonstrating the cardiovascular benefits of SGLT-2i (82–84), we did not find significant effect of SGLT-2i on the progression of cIMT. This may be related to the limited number of corresponding clinical studies, as we were only able to retrieve data on two SGLT-2i, ipragliflozin and tofogliflozin.

So far, metformin is still the first-line drug for people with diabetes (85). The results of our NMA also demonstrated a significant improvement in cIMT with the long-term administration of metformin. This finding aligns with a meta-analysis conducted by Chen et al. (86), their study also showed that metformin exhibited a significant impact on cIMT only when the intervention exceeded 12 months. Therefore, we speculate that longer duration of drug therapy is likely to result in a more pronounced therapeutic effect.

### Limitation

This study may represent the first NMA to compare and rank the effects of long-term use of different HDs on cIMT progression. This NMA may serve as a valuable reference for the utilization of cardiovascular protective drugs in individuals with diabetes or at risk of diabetes. However, the present NMA has some limitations. Firstly, limited number of studies and some small sample sizes may potentially affect the accuracy and applicability of the obtained results. Secondly, the research population in this NMA is based on type 1 diabetes patients, type 2 diabetes patients, pre-diabetes patients and coronary heart disease patients, which may lead to heterogeneity between the studies, so the interpretation of the results should be cautious. At last, we expect to see the performance of a variety of emerging HDs, such as DDP-4i of SGTL-2i, GLP-1 RA and PPARγ agonist. However, the available literature is currently limited. In conclusion, we anticipate to see more kinds of drugs and higher quality RCTs to verify our result in the future.

## Conclusions

We employed a Bayesian NMA to assess the impact of prolonged utilization of various HD methods on retarding the progression of carotid intima-media thickness (cIMT). Long-term administration of exenatide, alogliptin and metformin may be most effective in retarding the progression of cIMT.

## Data availability statement

The original contributions presented in the study are included in the article/supplementary material. Further inquiries can be directed to the corresponding author.

## Author contributions

QL: Conceptualization, Data curation, Formal Analysis, Methodology, Writing – original draft, Writing – review & editing. YY: Conceptualization, Data curation, Formal Analysis, Methodology, Writing – original draft, Writing – review & editing. YL: Data curation, Writing – original draft. QW: Data curation, Writing – original draft. XH: Formal Analysis, Writing – original draft. LL: Formal Analysis, Writing – original draft. XY: Data curation, Writing – original draft. CY: Data curation, Writing – original draft. SW: Conceptualization, Writing – review & editing.
