# Supplementary material for: Long-term effects of different hypoglycemic drugs on carotid intima-media thickness progression: a systematic review and network meta-analysis
Source: Front Endocrinol (Lausanne). 2024 May 31;15:1403606. doi: 10.3389/fendo.2024.1403606 (PMC11176463; doi:10.3389/fendo.2024.1403606)
Supplement: Supplementary file 1 [file DataSheet_1.docx]

Supplementary Material

# Appendix S1 Search strategy

# **MESH:**Carotid Intima-Media Thickness

**FREEDOM WORDS:**

Carotid Intima Media Thickness

Intima-Media Thickness, Carotid

# **MESH:**Atherosclerosis

**FREEDOM WORDS:**

Atheroscleroses

Atherogenesis

Atherogeneses

# **Hypoglycemic Agents**

(Gliclazide) or (glibenclamide) or (glimepiride) or (sulfonylurea) or (pioglitazone) or (thiazolidine) or (thiazolidinediones) or (sodium glucose co-transporter 2 inhibitor) or （sodium glucose co-transporter 2） or （sodium glucose co-transporter 2） or （ipragliflozin） or （dapagliflozin） or （luseogliflozin） or （tofogliflozin） or （canagliflozin） or （empagliflozin） or（ biguanides） or （metformin） or （acarbose） or （voglibose）or （miglitol） or （α-glucosidase inhibitor） or （α glucosidase inhibitor） or （mitiglinide） or （repaglinide） or （nateglinide） or （glinide） or （incretin） or （incretins dipeptidyl peptidase 4 Inhibitors） or （dipeptidyl peptidase 4 inhibitors） or（ saxagliptin） or （alogliptin） or （linagliptin） or （vildagliptin） or （sitagliptin） or（ teneligliptin） or（ anagliptin） or （trelagliptin） or （omarigliptin ）or （antidiabetic drugs） or （hypoglycemic medications） or （hypoglycemic agents）OR （gemigliptin） OR （Dipeptidyl-Peptidase IV Inhibitors） OR （Dipeptidyl Peptidase IV Inhibitors) OR (DPP-4 Inhibitor） OR （DPP 4 Inhibitor） OR （Inhibitor, DPP-4） OR （DPP-IV Inhibitor） OR （DPP IV Inhibitor） OR （Inhibitor, DPP-IV ）OR（ DPP-4 Inhibitors） OR （DPP 4 Inhibitors） OR（ DPP-IV Inhibitors） OR （DPP IV Inhibitors） OR（ Gliptin） OR （Dipeptidyl Peptidase 4 Inhibitor） OR （Dipeptidyl-Peptidase IV Inhibitor） OR （Dipeptidyl Peptidase IV Inhibitor） OR （Inhibitor, Dipeptidyl-Peptidase IV） OR （Dipeptidyl-Peptidase 4 Inhibitor） OR （Inhibitor, Dipeptidyl-Peptidase 4） OR （Dipeptidyl-Peptidase 4 Inhibitors） OR （Dipeptidyl Peptidase 4 Inhibitors） OR （Gliptins） OR （DPP4 Inhibitor） OR（ Inhibitor, DPP4） OR （DPP4 Inhibit）or （Glucagon Like Peptide 1） OR （GLP-1） OR （GLP 1） OR （Glucagon-Like Peptide-1） OR（ exenatide） OR（ Beinaglutide） OR（ liraglutide) OR (Loxenatide) OR( Semaglutide) OR (Albiglutide) OR (dulaglutide) OR (ipragliflozin) OR (Sodium-Glucose Transporter 2 Inhibitors) OR (Sodium Glucose Transporter 2 Inhibitors) OR(SGLT 2 Inhibitors) OR(SGLT2 Inhibitors)OR (Sodium-Glucose Transporter 2 Inhibitor) OR (Sodium-Glucose Transporter 2 Inhibitor) OR (SGLT2 Inhibitor) OR (Inhibitor, SGLT2 )OR (Gliflozins )OR (Gliflozin) OR (SGLT-2 Inhibitor) OR (Inhibitor, SGLT-2 )OR (SGLT 2 Inhibitor) OR( glipizide) OR (gliquidone)

# Appendix S2 **Results of sensitivity analyses**

| Omitted study | MD | 95% CI | | P |
| --- | --- | --- | --- | --- |
| Hanefeld，2004 (Acarbose) | -0.592 | -0.989 | -0.195 | 0.003 |
| Sidhu，2004 (Rosiglitazone) | -0.43 | -0.677 | -0.183 | 0.001 |
| Xiang，2005 (Troglitazone) | -0.61 | -1.004 | -0.216 | 0.002 |
| Hodis，2006 (Troglitazone | -0.587 | -0.984 | -0.189 | 0.004 |
| Hedblad，2007 (Rosiglitazone) | -0.485 | -0.822 | -0.149 | 0.005 |
| Hedblad2，2007 (Rosiglitazo~) | -0.613 | -1.006 | -0.22 | 0.002 |
| Mita，2007 (Nateglinide) | -0.599 | -0.995 | -0.203 | 0.003 |
| Eva，2009 (Rosiglitazone) | -0.608 | -1.003 | -0.213 | 0.003 |
| Koyasu，2010 (Acarbose) | -0.588 | -0.984 | -0.191 | 0.004 |
| Yamasaki，2010 (Pioglitazone) | -0.573 | -0.97 | -0.177 | 0.005 |
| Yasunari，2010 (Pioglitazone) | -0.607 | -1.001 | -0.214 | 0.002 |
| Saremi,2013 (Pioglitazone) | -0.591 | -0.988 | -0.193 | 0.004 |
| Patel,2013 (Acarbose) | -0.604 | -1 | -0.209 | 0.003 |
| Ishikawa，2014 (Sitagliptin) | -0.605 | -0.999 | -0.21 | 0.003 |
| Mita，2016 (Alogliptin) | -0.581 | -0.979 | -0.184 | 0.004 |
| Mita1，2016 (Sitagliptin) | -0.596 | -0.993 | -0.199 | 0.003 |
| Oyama1，2016 (Sitagliptin) | -0.612 | -1.006 | -0.218 | 0.002 |
| Katakami，2020 (Tofoglifozin) | -0.618 | -1.01 | -0.227 | 0.002 |
| Zhang，2020 (Exenatide) | -0.584 | -0.981 | -0.188 | 0.004 |
| Tanaka,2023 (Ipragliflozin) | -0.613 | -1.006 | -0.219 | 0.002 |
| Petrie,2017 (Metformin) | -0.61 | -1.005 | -0.216 | 0.002 |
| Meaney,2008 (Metformin) | -0.55 | -0.938 | -0.162 | 0.006 |
| Oyama,2008 (Acarbose) | -0.601 | -0.996 | -0.206 | 0.003 |
| Overall | -0.585 | -0.963 | -0.207 | 0.002 |
